# Maternity ward staff perceptions of exclusive breastfeeding in Finnish maternity hospitals: A cross-sectional study

**DOI:** 10.18332/ejm/134846

**Published:** 2021-05-31

**Authors:** Mervi Hakala, Pirjo Kaakinen, Maria Kääriäinen, Risto Bloigu, Leena Hannula, Satu Elo

**Affiliations:** 1 Oulu University Hospital, Oulu, Finland; 2 Research Unit of Nursing Science and Health Management, Faculty of Medicine, University of Oulu, Oulu, Finland; 3 Medical Research Center Oulu (MRC Oulu), Faculty of Medicine, University of Oulu, Oulu, Finland; 4 Medical Informatics and Statistics Research Group, Medical Imaging, Physics and Technology (MIPT) Research Unit, Faculty of Medicine, University of Oulu, Oulu, Finland; 5 Helsinki Metropolia University of Applied Sciences, Helsinki, Finland; 6 Lapland University of Applied Sciences, Lapland, Finland; 7 University of Oulu, Oulu, Finland

**Keywords:** questionnaire, infant, Baby-Friendly Hospital Initiative, exclusive breastfeeding, maternity ward staff, Step 6

## Abstract

**INTRODUCTION:**

This study aimed to describe exclusive breastfeeding (EBF, Step 6 of the Baby-Friendly Hospital Initiative) in Finnish maternity hospitals and identify factors that promote or limit EBF.

**METHODS:**

A cross-sectional study design was used, and data were collected from eight maternity hospitals in Finland during a 10-day period in May 2014. The staff completed questionnaires (n=1554) from separate work shifts. The data were analyzed using descriptive statistics, and chi-squared and Fisher’s tests. Responses to open-ended questions were analyzed using content analysis.

**RESULTS:**

Maternity ward staff reported that 72% (n=1105) of the infants were exclusively breastfed during their work shift. The strongest promoting factors of exclusive breastfeeding were: maternity ward staffs’ profession and education in breastfeeding counselling; multiparity; vaginal delivery; early skin-to-skin contact between mother and infant; initial breastfeeding after birth; rooming-in; and initial success of breastfeeding. The use of a nipple shield, the need for additional breastfeeding counselling, and infants’ blood tests were limiting factors to exclusive breastfeeding. Open-ended answers revealed that exclusive breastfeeding was mainly delayed because of medical issues for the mother or infant.

**CONCLUSIONS:**

Finnish maternity hospitals could improve exclusive breastfeeding rates by focusing attention and resources on breastfeeding counselling and evidence-based maternity care practices related to immediate care after birth, promoting vaginal delivery, rooming-in and availability of skilled counselling.

## INTRODUCTION

Maternity care hospitals play a key role in breastfeeding outcomes as breastfeeding rates can improve with evidence-based maternity practices and policies^1^. In 1991, the World Health Organization (WHO) and the United Nations International Children’s Emergency Fund (UNICEF) launched the Baby-Friendly Hospital Initiative (BFHI)^2,3^ as a global programme to curb the decrease in breastfeeding worldwide. The main objective of the BFHI is to promote, protect and support breastfeeding in facilities providing maternity and neonatal services. The programme also aims to ensure that every infant receives the best start for breastfeeding and recommends exclusive breastfeeding (EBF) for the first six months of life. The BFHI promotes the ‘Ten Steps to Successful Breastfeeding’, a practical guide that is distributed to maternity wards and facilities. In addition, the programme is integrated into the International Code of Marketing of Breast Milk Substitutes, another public health policy meant to protect breastfeeding^4^.

Step 6 of the BFHI guide states: ‘Do not provide breastfed newborns any food or fluids other than breast milk, unless medically indicated’^4^. WHO outlines the acceptable medical reasons for supplemental feeding of newborns at hospitals^5^. BFHI hospitals provide maternity ward staff with clear instructions regarding breastfeeding support; as a result, maternity ward staff at these hospitals work differently from those at non-BFHI hospitals^6^. Depending on the study, implementation of the BFHI guide is either partially^7^ or entirely^8^ responsible for the recent increase in EBF rates. In particular, the advice provided by the BFHI guide during these first days contributes both to successful breastfeeding at home^9^ and sustained, long-term EBF^10^. In this way, maternity ward staff are critical for encouraging and supporting breastfeeding^11^. Furthermore, a mother’s self-efficacy and confidence during the early postpartum period is strongly correlated with successful EBF and is particularly relevant for primiparas^12^.

EBF is beneficial for both infants and mothers; breast milk provides a perfectly-adapted nutritional supply and may also offer the infant specific personalized medicine with lifelong effects. If scaled up to a near-universal level, breastfeeding could, annually, prevent 823000 deaths in children under five years of age and 20000 deaths linked to breast cancer^1^.

Countries around the world have worked hard to improve EBF rates, however, the low rates of breastfeeding in high-income countries are cause for concern^1^. In Finland, EBF occurs during maternity hospital stays^13^ (66%), and although breastfeeding continuation rates are relatively high, the EBF rate at six months after birth is low, with only 9% of 6-month-old infants exclusively breastfed in 2019^14^. This is contrary to the global rate which has been steadily increasing; the global EBF rate for infants under six months of age is 43%^2^. Comparisons between countries demonstrate huge differences in the EBF rates at 6 months: Norway 35%, Sweden 16%, USA 27%, and UK <1%^1^. The current WHO global target is set at a rate of 50% of infants being exclusively breastfed during the first six months by 2025^15^.

Despite 28 years of existence of the BFHI, EBF rates at six months after birth are still far off-target in most high-income countries^16^ and the average EBF rate in Europe stands at 17%. Moreover, the difference between the global EBF rate and that of Europe is considerable. Although the rates of early breastfeeding initiation are very high in some European Union (EU) countries, EBF rates drop rapidly for infants aged 4–6 months and are very low at age 6 months^17^. Successful – and sustained – improvement of EBF rates in Europe is impossible without political and societal support. Unfortunately, modern society denies mothers a positive environment for EBF. Yet, breastfeeding mothers are not solely responsible for successful breastfeeding – the promotion of breastfeeding is a collective societal responsibility^16^.

Many maternity care practices strongly influence EBF rates^6^. Recent research showed that, during the postnatal period, any combination of the following events can increase EBF after discharge and during the subsequent months at home: skin-to-skin contact^18^, initiation of breastfeeding within an hour of birth^19^, rooming-in between mother and infant^20^, EBF during hospitalization^21^ and withholding a pacifier^22^. Use of combinations of various maternity care practices explains the variability in EBF rates better than the use of any single maternity care practice^6^. Previous studies recognize that supplemental feeding of breastfed infants is a common practice in Finnish maternity hospitals and may, at least partially, explain the low incidence of EBF later^23^. Studies also demonstrated that, during postnatal care in the hospital, mothers were least confident about breastfeeding along with supplemental feedings and about whether the infant is sufficiently fed^12^. Moreover, expectant parents lack adequate knowledge about breastfeeding^24^. Parents lacked awareness of the current recommendations about EBF, and many thought that supplemental feeding of the newborn was necessary^12^.

Several potential events during postnatal hospitalization may pose barriers to EBF: 1) infant appearing hungry after breastfeeding^25^, 2) infant admission to the neonatal intensive care unit (NICU)^26^, 3) low infant birth weight^27^, 4) birth by caesarean section^28^, 5) insufficient milk supply, 6) mother’s pain and discomfort^29^, 7) nipple and breast problems^30^, and 8) breastfeeding combined with supplemental feeding of formula^31^. During present-day hospitalizations, infants undergo many blood tests to measure blood glucose^32^, quantify bilirubin^33^ and/or detect infection^34^. These tests are crucial to improving infant health, but often lead to increased frequency of supplemental feedings.

Since EBF during postpartum hospitalization provides an important foundation for ensuring continued successful EBF at home^21^, a better understanding of the current state of maternity hospital postpartum care practices in Finland is required to remove potential inherent barriers to EBF. The continuous development in the healthcare sector means that there is always a need to research and develop better evidence-based practices. This study aimed to describe EBF (Step 6 of the Baby-Friendly Hospital Initiative) in Finnish maternity hospitals and identify factors that promote or limit EBF.

## METHODS

### Participants and data collection

In 2014, there were 29 maternity hospitals in Finland^35^. These maternity hospitals were divided into four different size classes to provide homogeneous subsets. Eight participating hospitals in Finland were chosen using stratified random sampling^36^ such that the study included two hospitals that represented each of the following hospital categories defined by the National Institute for Health and Welfare: under 750 deliveries, 750–1500 deliveries, over 1500 deliveries, and university hospital^35^. A total of two of the eight study hospitals had a BFHI certificate. Each maternity hospital provided one contact person who was responsible for sharing information about the study with the staff.

Data were collected from maternity ward staff during a 10-day period in May 2014 and 279 births occurred during the data collection period. A total of 509 mothers gave birth during this period. All of the mothers who had given birth during this period were asked to participate in the study, with 55% (n=279) participating in the study. Therefore, midwives or nurses completed a questionnaire for the same infants and mothers several times during the research period, with f representing the number of completed questionnaires. The staff completed questionnaires for each infant they attended during their work shift. The completed number of questionnaires were 1554 for 279 infants.

**Figure 1:**
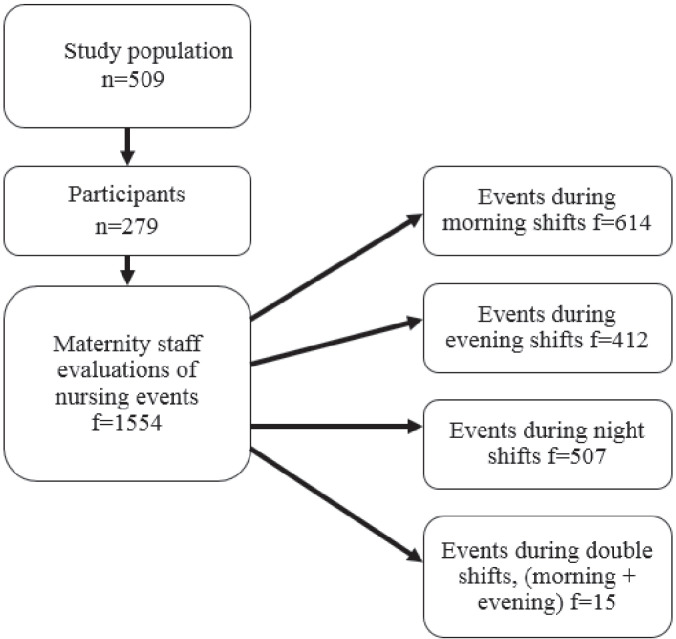
Schematic showing the study structure and the questionnaire responses over various shifts.

Figure 1 outlines the structure of the study and lists the number of questionnaire responses collected from different work shifts during the study period. In the context of this study, EBF means that the infant does not receive any food other than their mother’s breast milk.

### Questionnaire

The questionnaire employed in this study asked mother– infant pairs and nurses about factors which have been previously reported to be significantly connected to EBF. The questionnaire for maternity ward staff was developed based on previous studies of the BFHI with the help of expert panels^37,38^. It consisted of open-ended, dichotomous (yes/ no) or multiple-choice questions (Table 1) and was presented in two languages: Finnish and Swedish. Content validity of the questionnaire was evaluated by three different expert panels: committee members from the Federation of Finnish Midwives (n=7), midwives (n=6), and experts at developing questionnaires to fit the requirements of nursing science (n=3). Few changes were made to the questionnaire after the evaluation, and the questionnaire underwent a pilot study.

**Table 1:** Content and type of questions in the maternity ward staff questionnaire

*Type of question*	*Topic pertaining to maternity ward staff*
**Open-ended**	Hospital (name, size, Baby-Friendly status), age, years of work experience, staff responsibilities during shift, reasons for skin-to-skin contact, barriers to full rooming-in, amount of supplementation, blood test times, bed-day, reasons for supplementation, reasons for blood test (for infant)
**Multiple-choice**	Occupation, work shift, duration that full rooming-in did not occur, quality of supplementation, method of supplementation
**Dichotomous**	Completion of training in breastfeeding counselling, sufficient knowledge of breastfeeding counselling, opinion of full rooming-in, implementation of full roomingin, adequate time to provide breastfeeding counselling, success of breastfeeding, need for breastfeeding counselling, need for nipple shield, need for supplementation, need for blood test (for infant), implementation of skin-to-skin contact

Pilot study data were collected from three maternity hospitals in Finland for one week in April 2014 using both Finnish and Swedish questionnaires. The evaluations resulted in a final version of the questionnaire, which included 27 questions: nine background questions – on hospital (name, size, Baby-Friendly status), age, occupation, years of work experience, work shift, staff responsibilities during shift, completion of training in breastfeeding counselling, sufficient knowledge of breastfeeding counselling, and opinion of full rooming-in; ten structured questions – success of breastfeeding, need for supplemental food, blood tests, nipple shield use, breastfeeding counselling, sufficiency of time for breastfeeding counselling, frequency of supplemental food and blood tests, and quality and mode of supplemental food; and two open-ended questions concerning breastfeeding – reasons for supplemental food and blood tests. Other items on the questionnaire covered skin-to-skin contact and rooming-in^39,40^ (Table 1).

### Ethical issues

All participating hospitals granted permission to perform the study upon request. Requests for approval of study conduct from the Regional Ethics Committees of the Northern Ostrobothnia Hospital District and the Turku Clinical Research Centre resulted in unambiguous responses from both committees stating that the study did not require ethics committee approval according to the Medical Research Act (1999/488)^41^. Hence, permission from each participating hospital was sufficient.

Maternity ward staff participation in the study was voluntary. Staff were informed about the study by an internal contact person both orally and via a cover letter. All participating staff (from the maternity ward and delivery room) gave informed oral consent to participate based on the information provided. The main researcher did not establish a register of participating staff, and the study was designed to ensure the anonymity of every participant^42^. The questionnaires and the data they produced were coded such that the researchers could handle the data without losing information.

### Data analysis

Data analysis was conducted using SPSS Statistics for Windows (version 24.0, IBM, Armonk, NY). The data were examined using descriptive statistics (frequencies, percentages, median). Chi-squared and Fisher’s tests were used to compare the differences between groups. The level of statistical significance was set at p<0.05^36^. Participants with missing data were excluded by listwise deletion.

The answers to open-ended questions were subjected to content analysis^43-45^, an appropriate means of analysis since qualitative answers describe phenomena better than numeric answers^44^. These answers were typically short, comprising only a few words or short paragraphs, thus profound content analysis was impossible to perform. Answers to open-ended questions were described using q, for the number of similar answers, as identified through content analysis.

## RESULTS

### Participant characteristics

There were more than a hundred maternity ward staff members working in these eight units during the data collection period. An unknown number of them completed the total of 1554 questionnaires of separate work shifts of infants they attended. The mean age of maternity ward staff was 43 years (range 20–64; median 45). Their mean duration of work experience was 16 years (range 0–37; median 14). Most of the maternity ward staff (67%) were midwives, and a majority (97%) were trained in breastfeeding counselling. Maternity ward staff cared for an average of 4.5 infants per shift (range 0–29; median 4) (Table 2).

**Table 2:** Background information on maternity ward staff filling the questionnaires (Completed questionnaires = 1554)

*Background*	f*	*%*	*Range*
**Education level**			
Midwives	961	66.7	
Practical nurses	404	28	
Registered nurses	76	5	
Nursing students	5	0.3	
**Age** (years)			20 – 64
20–30	320	21	
31–40	349	23	
41–50	318	20	
51–60	499	32	
>60	62	4	
**Years working as a nurse**			0 – 37
0–10	638	43	
11–20	272	18	
21–30	380	25	
31–40	209	14	
**Shift**			
Morning	614	40	
Evening	412	26	
Night	507	33	
Double (morning and evening)	15	1	
**Breastfeeding counselling training completed**			
Yes	1502	97	
No	52	3	
**Staffs’ responsibility (infants/nurse)**			0 – 29
0–5	1132	74	
6–10	348	23	
≥11	50	3	

* Number of completed questionnaires.

Most of the mothers (n=272) who participated to this study were primiparous (46%, n=125), with II-parturient mothers (35%, n=96) being the second most common group. The research also included some grand multiparous mothers (range 1–16). Most of the mothers gave birth vaginally (normal or assisted vaginal delivery; 86%, n=232), while caesarean section was performed in 14% (n=38) of cases. The mothers were, on average, in week 39 of their pregnancy during childbirth (median 39, range 30–42).

### Implementation of exclusive breastfeeding (EBF) and the factors that promote or limit EBF and the factors that promote or limit EBF

Maternity ward staff filled in the questionnaire during each shift and marked down whether the infant had received supplemental food. Maternity ward staff reported that 72% (n=1105) of the infants were exclusively breastfed during their work shift. The supplemental food was either formula (56%, n=236) or donor breast milk (44%, n=183). Some infants also received their own mother’s pumped breast milk (n=78). Most infants who were given supplemental food received it once per shift (43%, n=178), while fewer infants received supplemental food twice per shift (29%, n=117), or three or more times per shift (28%, n=116). About a third of infants (36%, n=560) received a blood test during hospitalization. Over half of the reported blood tests were performed once per shift (65%, n=347) while far fewer tests were performed three or more times per shift (7%, n=38).

Several factors connected to staff, mothers and infants were found to significantly promote or limit EBF. For example, the following staff characteristics were found to decrease EBF: staff age (p=0.039); nurse’s education level (p=0.003); and lack of training in breastfeeding counselling (p=0.001). The highest rates of EBF were identified among staff aged >60 years, while staff aged 41–50 years were linked with the lowest rates of EBF. The lowest rates of EBF were associated with shifts during which registered nurses took care of infants, whereas the shifts in which nursing students or midwives (74%, n=697) took care of infants revealed the highest rates of EBF. Having received education in breastfeeding counselling increased the rate of EBF to three-quarters relative to only half of infants in cases when staff had not received this education. Table 3 shows the factors that significantly influenced the rate of EBF.

**Table 3:** Background factors connected with EBF, according to labor room and maternity ward staff

*Background factor*	*EBF*		*non-EBF*		*p*
	*n*	*%*	*n*	*%*	
**Age of maternity ward staff**					0.039
20–30	234	74	82	26	
31–40	253	73	95	27	
41–50	204	65	110	35	
51–60	361	73	132	27	
>60	48	79	21	13	
**Profession of staff**					0.003
Midwives	697	74	249	26	
Practical nurses	287	71	117	29	
Registered nurses	40	53	47	35	
Nursing students	4	80	1	20	
**Completion of training in breastfeeding counselling**					0.001
Yes	1079	73	408	27	
No	26	50	26	50	
**Mother’s parity**					<0.001
Primipara	486	67	235	33	
Multipara	598	76	188	24	
**Mode of childbirth**					<0.001
Vaginal birth	935	74	325	26	
Caesarean section	144	60	96	40	
**Days of hospitalization**					<0.001
0 (birth)	170	81	40	19	
1 day after birth	364	74	127	26	
2 days after birth	298	67	146	33	
≥3 days after birth	163	62	99	38	

EBF: exclusive breastfeeding.

A mother’s characteristics which decreased EBF included: primiparity (p<0.001); birth by caesarean section (p<0.001); the number of hospitalization days (p<0.001) (Table 3); receipt of additional breastfeeding counselling (p<0.001); and use of a nipple shield (p<0.001). A third of the primiparous mothers did not exclusively breastfeed, which can be compared to an EBF rate of 75% among multiparous mothers. Infants delivered through caesarean section were less (60%) likely to be exclusively breastfed than infants delivered vaginally (74%). Moreover, the length of an infant’s hospitalization was positively connected to how much supplemental food they received. Receiving additional breastfeeding counselling was negatively linked with EBF (78% vs 66%), and the use of a nipple shield decreased EBF (56% vs 76% with and without a nipple shield, respectively). Table 4 presents the maternity care practices that significantly influenced EBF.

**Table 4:** Maternity care practices connected with EBF, according to labor room and maternity ward staff

*Classification*	*Influencing factor*	*EBF*		*non-EBF*		*p*
		*n*	*%*	*n*	*%*	
**Skin-to-skin contact (SSC)**	**Early SSC between mother and infant**					
	Day I Yes	163	76	51	24	0.001
	No	12	40	18	60	
	Day II Yes	113	73	42	27	<0.001
	No	7	35	13	65	
	**Starting age of early SSC**					
	Day II ≤5 minutes	105	75	36	25	0.013
	>5 minutes	14	50	14	50	
	**Implementation of SSC at the ward**					
	Yes	626	74	220	26	
	No	453	69	208	31	
**Initial breastfeeding**	**Success of initial breastfeeding**					
	Day I Yes	145	80	36	20	<0.001
	Try to suck	19	50	19	50	
	No	6	40	9	60	
	Day II Yes	102	80	26	20	<0.001
	Try to suck	14	45	17	55	
	No	3	30	7	70	
	**Starting age of initial breastfeeding**					
	Day II ≤1 hour	104	77	31	23	0.014
	>1 hour	13	52	12	48	
	**Length of initial breastfeeding**					
	Day II ≤1 hour	85	70	37	30	0.009
	>1 hour	28	93	2	7	
**Rooming-in**	**Implementation of rooming-in**					<0.001
	Full rooming-in	1055	75	355	25	
	Partial rooming-in	49	39	78	61	
	**Duration of unrealized rooming-in**					0.008
	<1 hour	39	61	25	39	
	≥1 and <3 hours	19	44	24	56	
	≥3 and <5 hours	6	27	16	73	
	≥5 hours	13	33	27	67	
**EBF, breastfeeding**	**Success of breastfeeding**					<0.001
	Yes	992	76	312	24	
	No	99	47	111	53	
	**The need for breastfeeding counselling**					<0.001
	Yes	476	66	247	34	
	No	623	78	178	22	
	**Nipple shield use**					<0.001
	Yes	169	56	131	44	
	No	927	76	293	24	
	**Frequency of supplemental food**					0.038
	1 time	12	7	166	93	
	2 times	2	2	114	98	
	≥3 times	2	2	114	98	
	**The need for a blood test**					<0.001
	Yes	338	61	219	39	
	No	762	78	213	22	
	**Frequency of blood tests**					<0.001
	1 time	235	68	109	32	
	2 times	78	53	68	47	
	≥3 times	12	32	25	68	

EBF: exclusive breastfeeding.

The factors related to infants which decreased EBF were: infant was not rooming-in with the mother (p<0.001); and time spent away from the mother’s room (p=0.008). Infants who roomed-in day-and-night showed a 75% EBF rate, which can be compared with the EBF rate of 39% when rooming-in was not practiced. Moreover, the amount of time that infants were out of the mother’s room was positively linked with the amount of supplemental food they received. For example, infants who were out of the mother’s room for three to five hours per day showed EBF rates of 27%, which can be compared to an EBF rate of 61% when the infant was out of the room for under one hour. Other factors that decreased EBF rate were frequency of supplemental food (p=0.038), the need for blood tests (p<0.001), and repeated encounters with blood tests (p<0.001). The amount of supplemental food that an infant received was inversely related with the EBF rate. In addition, the EBF rate among infants who needed a blood test (61%) was noticeably lower than the EBF rate among infants who did need blood tests (78%). Moreover, the number of blood tests an infant required was negatively related to the EBF rate. For example, the EBF rate among infants who needed three or more blood tests was 32%, which can be compared to an EBF rate of 68% among infants that only needed one blood test. Problems with breastfeeding (p<0.001) also decreased the EBF rate. Infants who succeeded in sucking demonstrated noticeably higher EBF rates (76%) than infants who did not suck well (47%). In contrast, skin-to-skin contact at the postnatal ward (p=0.021) reduced occurrence of supplemental food. Skin-to-skin contact with mother increased EBF (74% vs 69%) (Table 4)

Additional factors that increased EBF included: early skin-to-skin contact between mother and infant (Day I: p=0.001; Day II: p<0.001); starting skin-to-skin contact with the infant as soon as possible (p=0.013), success of initial breastfeeding (Days I and II: p<0.001), age of under an hour at initial breastfeeding (Day II: p=0.014), and duration of initial breastfeeding of over one hour (Day II: p=0.009) in the labor ward. In cases of early skin-to-skin contact between mother and infant, 76% and 73% of the infants did not receive supplemental food at the maternity ward on Days I and II of hospitalization, respectively. Infants who did not experience skin-to-skin contact with the mother soon after delivery were less likely to be exclusively breastfed (Day 1: 69%; Day II: 40%). Infants whose early skin-to-skin contact started less than five minutes after birth received less supplemental food at the maternity ward on Day II of hospitalization. Infants who succeeded well at initial breastfeeding after birth also received less supplemental food than infants who tried to suck or did not suck (Day I: 53%, n=28; Day II: 59%, n=24). Infants who started initial breastfeeding less than one hour after birth received less supplemental food on Day II of hospitalization than infants who started to suck for the first time more than an hour after birth (Day II: 48%, n=12). Infants whose initial breastfeeding lasted for over one hour received less supplemental food on Day II of hospitalization than infants who sucked for less than one hour (Day II: 30%, n=37) (Table 4)

Maternity ward staff provided reasons for supplemental food in response to open-ended questions, and many gave previously specified reasons. The most commonly reported reasons for supplemental food (Q=835) were: a hungry and unsatisfied infant (q=160), prematurity, jaundice, and excessive weight loss (q=149), low blood glucose or glucose measurements (q=134), insufficient milk supply (q=118), infant would not suck (q=98), mother’s health and other problems (q=68), infant problems (q=57), mother’s desire (q=47), and medical reasons (q=4).

## DISCUSSION

This study aimed to describe EBF (Step 6 of the BFHI programme) in Finnish maternity hospitals, as well as identify factors that promote or limit EBF. The results, which are based on data collected in 2014, revealed that most Finnish mothers started to breastfeed during hospitalization; as such, most infants received only their own mother’s breast milk in maternity hospitals. However, this study’s findings showed that a third of infants in Finnish maternity hospitals are commonly given supplemental food, confirming the findings of a previous study in Finland^12^. However, we cannot be sure that 72% of the infants were exclusively breastfed from birth to discharge, only that they were exclusively breastfed during those shifts. There might be some missing notes from an individual infant. Most participating staff members in this study referred to supplemental food as formula. Moreover, some participating maternity hospitals were unable to collect and use donated breast milk for supplemental food. The study population of participants reflected Finnish national statistics well^46^ with regard to background characteristics like mother’s parity, age, and mode of childbirth. Thus, the findings of the study may be generalized to depict the state of EBF^35^.

In this study, the education level of maternity ward staff and lack of training in breastfeeding counselling for some staff members may have negatively affected EBF rates. One important means of increasing EBF rates would be to train all maternity hospital staff that care for women and infants in provision of breastfeeding support (like the WHO 20-hour breastfeeding counselling course). Midwifery education in Finland includes this course, which explains why the staff educational background significantly influences the EBF rate. The maternity ward should be staffed by professionals who are competent in counselling on EBF and always available to assist mothers. A previous study^47^ indicates that training medical doctors in breastfeeding can also increase EBF rates among the women they care for. This opportunity for development for medical doctors was suggested through the new Finnish National Action Program for the Promotion of Breastfeeding 2018–2022^46^. Maternity ward staff must remember to offer breastfeeding counselling to both primiparas and multiparas as each infant is unique and mothers may need individualized assistance. Additionally, both mother and infant have significant roles in successful breastfeeding. The results of this study suggest that maternity ward staff and the hospital’s breastfeeding culture are critical early factors affecting the journey towards successful and sustained EBF.

As mentioned above, this study found that several mothers’ background factors decreased EBF, including birth by caesarean section and giving birth for the first time. Other studies have identified the same relationships^18,48^. It is important to note that psychological factors are highly predictive of EBF outcomes. Primiparas need special support to acquire early postpartum self-efficacy^49^. Moreover, some primiparous mothers will need more counselling, encouragement and advice from maternity ward staff to establish self-confidence for breastfeeding and breast milk production. Supplemental food of an infant during the first days immediately after birth can negatively affect the mother as she may feel that she has insufficient breast milk to feed her infant. Self-efficacy is essential for mothers as it can influence the success of EBF both at the maternity ward and at home^12^. When a mother succeeds at breastfeeding her first child, she will likely be more confident when breastfeeding subsequent children. This study also revealed that the use of a nipple shield is more widespread in the population of non-exclusively breastfed infants. Kronborg et al.^50^ found that nipple shield use increased the risk of earlier cessation of EBF. These findings suggest that maternity ward staff should be aware of these risks to EBF and exercise caution in offering nipple shields to primiparas and mothers who have given birth by caesarean section. Alarmingly, study findings revealed that increased duration of a mother’s hospital stay was negatively connected to successful EBF, a result which has also been reported by Schmied et al.^11^. Mothers today spend less time – ranging from several hours to a couple of days – in the hospital than before, yet this period is crucial for a successful start to breastfeeding. Indubitably, a longer hospital stay could be explained by postpartum problems for the mother or infant. Nevertheless, all maternity ward staff should be vigilant and proactive in ensuring the success of EBF. Time spent at the hospital should provide mothers with the support they need to continue breastfeeding at home.

This study identified positive connections between EBF and the implementation of other BFHI steps like early skin-to-skin contact, breastfeeding immediately after birth, and rooming-in. The results also showed that the time that mothers and infants spend together – both day and night – is positively connected to EBF success, as corroborated by previous research^21^. In addition, this study demonstrated that skin-to-skin contact at the maternity ward positively influenced a mother’s tendency to practice skin-to-skin at later time points, a finding which is supported by previous research^51^. These findings support the ideas that implementing the BFHI steps can increase EBF during hospitalisation^6^, and that they increase the likelihood of early and continued EBF. The effectiveness of the BFHI programme is evident worldwide and, in Finland, many maternity hospitals are working towards getting the BFHI certificate. The state of BFHI certification in other Scandinavian countries was noticeably better than in Finland, but the proportion of BFHI-certified hospitals across the Nordic countries has decreased^18^. The Finnish maternity hospitals that participated in this study implemented Step 6 of the BFHI programme to a satisfactory extent, despite only two of them holding BFHI certificates. A likely explanation for this phenomenon is that Finnish maternity care culture aims to implement early skin-to-skin contact, breastfeeding immediately after birth, rooming-in, and EBF. Surprisingly, the two participating BFHI-certified hospitals did not significantly differ from the other studied hospitals in terms of EBF rates. A previous study^52^ argued that in a country with high breastfeeding initiation rates, the BFHI certificate itself makes no meaningful difference in breastfeeding continuation or exclusivity rates. This may explain why no significant between-hospital differences in EBF rates were observed. In these countries, the number of BFHI practices implemented during the mother’s postpartum care matters more.

Supplemental food is generally considered acceptable when the infant shows signs of inadequate milk intake which, if not addressed, may lead to excessive weight loss, jaundice, and low blood glucose. Previous research suggested that mothers should be informed of the reasons for supplemental food to prevent any later hindrances to EBF^35^. In this study, the maternity ward staff reported that supplemental food was most commonly given because of a hungry and unsatisfied infant; another study also identified this as the main reason for providing infants with supplemental food^10^. Maternity ward staff should be able to explain to mothers and parents that infants normally cluster feed around 24 hours postpartum as this behavior helps to stimulate the onset of mature milk. It is likely that some of the infants included in this study exhibited this cluster feeding and – for this reason – did not require supplemental food. Additional reasons for supplemental feeding were infant prematurity, jaundice or excessive weight loss. Infant prematurity is a previously identified barrier to EBF implementation^53^. The need for blood glucose measurements in infants was another impediment to EBF. The infants of mothers with gestational diabetes will require blood glucose measurements and, in 2014, 16% of pregnant Finnish women had gestational diabetes; the prevalence is increasing^35^. The contemporary increase in medicalized postnatal care today translates to infants being subjected to more blood tests during hospitalization than before, resulting in a negative impact on EBF rates. Maternity ward staff must always consider whether treatment is necessary and justified for a mother or infant so that the staff can provide adequate, evidence-based treatment at the correct time^54^. Many of the infants included in this study received blood glucose tests followed by supplemental food to increase their blood glucose level. However, and noteworthily, Tozier^55^ found that infant blood glucose values do not improve after formula intake. Participating maternity ward staff also cited insufficient milk production as an impediment to continued EBF, corroborating the findings of another study^19^. According to participating maternity ward staff, infants were given supplemental food when they had health issues or other problems, or when the mother had breastfeeding problems^56^. Maternity ward staff carefully specified the reasons for supplemental food, i.e. due to the condition of the infant or mother, and most were medical reasons. Nevertheless, this study demonstrated that supplemental food was also provided for non-medical reasons. This is a phenomenon that requires further research, i.e. what are the determinants of supplemental food for non-medical reasons.

UNICEF clearly stated that ‘Breastfeeding is not a onewoman job; it requires government leadership and support from families, communities, workplaces, and the health system to really make it work’^57^. In Finland, the recently published National Action Program for the Promotion of Breastfeeding 2018–2022 presents several useful strategies for successful breastfeeding. It also identifies a need for systematic annual monitoring, and close co-operation between public health services and maternity hospitals^46^. In fact, one of the targets of this promotion is to transform Finland into a world leader in breastfeeding.

### Limitations

This study had certain inherent limitations and, thus, the results presented should be interpreted with caution. First, because the maternity ward staff questionnaire was anonymously completed after every shift, it is impossible to know which staff member completed each questionnaire. One staff member completing a large proportion of the questionnaires could introduce a fair amount of bias into the research. However, since the staff worked in three rotating shifts, such bias is unlikely. In addition, missing data for an individual infant could not be retrieved, therefore the circumstances surrounding EBF for that particular mother–infant pair may be imprecisely reflected in the study results. We were unable to perform multivariable logistic regression analyses, which would have provided adjusted results instead of crude results. The data were collected in 2014; as such, more research is needed to describe the current situation and determine if maternity care practices have changed.

## CONCLUSIONS

The rates of EBF in Finnish maternity wards are relatively high but could still be improved. Several factors related to maternity staff, mothers and infants were found to promote or limit EBF. For example, counselling of breastfeeding seems positively promote EBF. On the other hand, delivery by caesarean section, primiparity, nipple shield use, insufficient breastfeeding counselling and longer duration of hospitalization were all negatively linked with EBF. The presented findings also revealed that early skin-to-skin contact, breastfeeding immediately after birth, and rooming-in can increase EBF during hospitalization. An infant’s need for blood tests, e.g. due to a mother’s gestational diabetes, was found to increase the risk of EBF failure. Based on the presented findings, EBF rates could be improved by providing all maternity ward staff with breastfeeding education, improving evidence-based hospital practices related to immediate care after birth, emphasizing the negative aspects of delivery by caesarean section, implementing rooming-in, and ensuring that every new mother can get breastfeeding guidance.
